# Regional High Iron in the Substantia Nigra Differentiates Parkinson’s Disease Patients From Healthy Controls

**DOI:** 10.3389/fnagi.2019.00106

**Published:** 2019-05-27

**Authors:** Kiarash Ghassaban, Naying He, Sean Kumar Sethi, Pei Huang, Shengdi Chen, Fuhua Yan, Ewart Mark Haacke

**Affiliations:** ^1^Department of Radiology, Wayne State University, Detroit, MI, United States; ^2^Department of Radiology, Ruijin Hospital, Shanghai Jiao Tong University School of Medicine, Shanghai, China; ^3^Magnetic Resonance Innovations, Inc., Bingham Farms, MI, United States; ^4^Department of Neurology & Institute of Neurology, Ruijin Hospital, Shanghai Jiao Tong University School of Medicine, Shanghai, China

**Keywords:** brain, iron, magnetic resonance imaging, Parkinson’s disease, substantia nigra, deep gray matter, quantitative susceptibility mapping, relaxometry

## Abstract

**Background:** Iron is important in the pathophysiology of Parkinson’s disease (PD) specifically related to degeneration of the substantia nigra (SN). Magnetic resonance imaging (MRI) can be used to measure brain iron in the entire structure but this approach is insensitive to regional changes in iron content.

**Objective:** The goal of this work was to use quantitative susceptibility mapping (QSM) and R2^∗^ to quantify both global and regional brain iron in PD patients and healthy controls (HC) to ascertain if regional changes correlate with clinical conditions and can be used to discriminate patients from controls.

**Methods:** Susceptibility and R2^∗^ maps of 25 PD and 24 HC subjects were reconstructed from data collected on a 3T GE scanner. For the susceptibility maps, three-dimensional regions-of-interest (ROIs) were traced on eight deep gray matter (DGM) structures and an age-based threshold was applied to define regions of high iron content. The same multi-slice ROIs were duplicated on the R2^∗^ maps as well. Mean susceptibility values of both global and regional high iron (RII) content along with global R2^∗^ values were measured and compared not only between the two cohorts, but also to susceptibility and R2^∗^ baselines as a function of age. Finally, clinical features were compared for those PD patients lying above and below the upper 95% regional susceptibility-age prediction intervals.

**Results:** The SN was the only structure showing significantly higher susceptibility in PD patients compared to controls globally (*p* < 0.01) and regionally (*p* < 0.001). The R2^∗^ values were also higher only in the SN of PD patients compared to the healthy cohort (*p* < 0.05). Furthermore, those patients with abnormal susceptibility values lying above the upper 95% prediction intervals had significantly higher united Parkinson’s diagnostic rating scores. R2^∗^ values had larger errors and showed larger dispersion as a function of age than QSM data for global analysis while the dispersion was significantly less for QSM using the RII iron content.

**Conclusion:** Abnormal iron deposition in the SN, especially in RII areas, could serve as a biomarker to distinguish PD patients from HC and to assess disease severity.

## Introduction

Parkinson’s disease (PD) is believed to be the second most common neurodegenerative disease in developed countries (Shulman and De Jager, 2009). Research has shown that the substantia nigra (SN) is one of the most important structures playing a vital role in the pathophysiology of PD patients (Ghassaban et al., 2018). Neuronal loss and lack of dopamine content in this midbrain nucleus generally lead to movement disorders in these patients (Wang et al., 2016; Martin-Bastida et al., 2017). The loss of neuromelanin in particular has been thought to lead to an increase in iron content in the SN which has been implicated in a number of PD studies (Castellanos et al., 2015; Huddleston et al., 2017; Langley et al., 2017). In fact, the SN seems to be the only reliable brain structure through which a meaningful relationship with neuronal loss has been found (Ghassaban et al., 2018). Since the onset of PD is generally late and brain iron levels tend to increase as a function of age in deep gray matter (DGM) structures even under normal conditions, (Hallgren and Sourander, 1958; Li et al., 2014; Liu et al., 2016) it is important to account for these age-dependent changes (Acosta-Cabronero et al., 2017).

A number of magnetic resonance imaging (MRI) techniques can be used to quantify iron content in the human body. Conventionally, R2 and R2^∗^ relaxation rate mapping along with phase information have been utilized to measure iron deposition in different regions of the human body *in vivo* (Ghassaban et al., 2018). However, one of the most popular approaches today is the use of quantitative susceptibility mapping (QSM); a post-processing technique that generates susceptibility maps using phase information and, unlike other conventional quantification techniques, is independent of imaging parameters such as geometry, echo time, spatial resolution, field strength, and signal-to-noise ratio (SNR) (Haacke et al., 2015). QSM also appears to have the greatest reliability and robustness compared to other MR-based *in vivo* methods (Haacke et al., 2015; Du et al., 2016; Langkammer et al., 2016). Specifically, in terms of consistency, QSM has been shown to have high repeatability and less variability compared to R2^∗^ (Feng et al., 2018).

Liu et al. (2016) investigated a cohort of 174 healthy adults using QSM with the purpose of assessing the effects of normal aging on the iron levels in seven DGM structures. In addition to their evaluation of mean susceptibility from the entire 3D region covered by each nucleus as a function of age (also known as the global analysis) in the basal ganglia and midbrain, they introduced a new age- and structure-dependent high iron susceptibility-age baseline (also known as the regional analysis). The regional analysis appeared to be more robust and sensitive to age-related changes compared to the global analysis (Liu et al., 2016). Furthermore, by applying the same methodology, Ghassaban et al. (2018) established the global and regional susceptibility-age baselines for the dentate nucleus using 81 healthy adults. Therefore, we hypothesized that this regional analysis may also be more sensitive to changes in iron for PD patients.

In this study, using QSM and R2^∗^ techniques, we compare the iron content in the DGM structures between a cohort of PD patients and a group of healthy controls (HC). Additionally, using QSM maps we investigate the iron deposition rates of PD patients compared to the corresponding global and regional normal baselines established by Liu et al. (2016) and Ghassaban et al. (2018) Similarly, R2^∗^ maps are used to compare global measurements to those of the healthy population established by Li et al. (2014). Also, susceptibility measurements in terms of increased iron deposition are compared to the clinical status of PD patients. Finally, the QSM data are compared directly to the R2^∗^ across the different DGM nuclei using the HC data. This study could potentially pave the way for developing future iron-based diagnostic studies and better understanding the etiology of PD.

## Materials and Methods

### Data Collection

This study was approved by the local ethics committee at Ruijin Hospital and all subjects signed consent forms. A total of 49 subjects were evaluated: 25 PD patients (61.8 ± 6.4 years old) and 24 HC subjects (63.4 ± 8.0 years old). All of the PD patients were recruited from local movement disorder clinics. The inclusion criteria were: (1) a diagnosis of idiopathic PD, (2) Mini-Mental State Exam (MMSE) score equal to or more than 24, and (3) Hoehn and Yahr (H&Y) scale of one through three as patients with higher scores had more severe symptoms and would have trouble staying still in the magnet for the duration of the scans. The exclusion criteria were: (1) symptoms of secondary or atypical parkinsonism, or (2) a history of cerebrovascular disease, seizures, brain surgery, brain tumor, moderate-to-severe head trauma, or hydrocephalus, or (3) treatment with antipsychotic drugs or with any other drug possibly affecting clinical evaluation. Data were collected using a 16 echo, gradient echo imaging sequence on a 3T GE Signa HDxt from an eight-channel receive-only head coil with the following imaging parameters: TE1 = 2.69 ms with ΔTE = 2.87 ms, TR = 59.3 ms, pixel bandwidth = 488 Hz/pixel, flip angle = 12°, slice thickness = 1 mm, matrix size = 256 × 256, and an in-plane resolution of 0.86 × 0.86 mm^2^.

### Data Processing

#### QSM Processing

**Figure 1:**
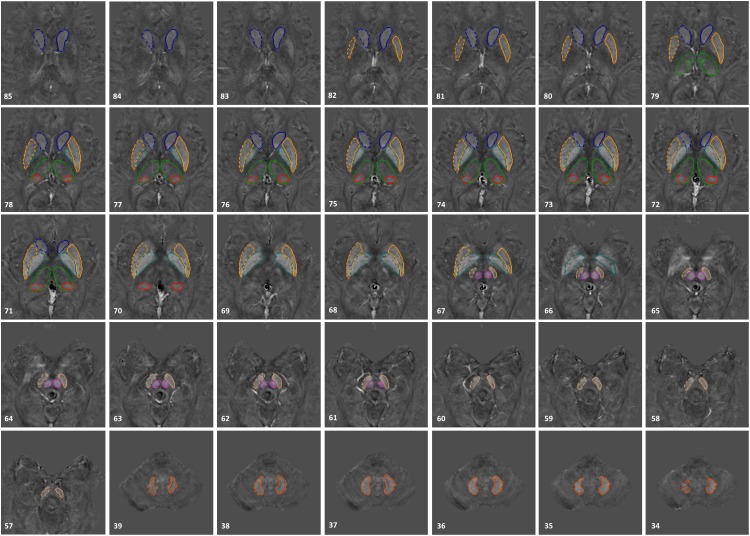
3D regions of interest (ROIs) traced on susceptibility maps of a 65-year-old male. Structures include the head of caudate nucleus (CN), globus pallidus (GP), putamen (PUT), thalamus (THA), pulvinar thalamus (PT), red nucleus (RN), substantia nigra (SN), and dentate nucleus (DN). The numbers in the lower left corner represent the slice numbers from 132 slices collected in this example.

The susceptibility maps were created using the first eight echoes and were reconstructed for each echo individually using SMART v2.0 (MRI Institute for Biomedical Research, Bingham Farms, MI, United States) followed by a weighted average of the resultant QSM images based on their SNRs. Only 8 echoes were used because of severe frontal signal loss at echo times longer than roughly 20 ms. The reconstruction steps included the brain extraction tool (BET) to segment only the brain tissue using the fourth echo magnitude data, (Smith, 2002) quality guided 3D phase unwrapping algorithm (3DSRNCP) for phase unwrapping, (Abdul-Rahman et al., 2007) sophisticated harmonic artifact reduction for phase data (SHARP) for background field removal with a threshold of 0.05 and a deconvolution kernel of 6, (Schweser et al., 2011) and a truncated k-space division (TKD) approach (threshold = 0.1) referred to as susceptibility weighted imaging and mapping (SWIM) for inverse filtering (Haacke et al., 2010). The DGM nuclei included in this study were: the head of the caudate nucleus (CN), putamen (PUT), globus pallidus (GP), thalamus (THA), pulvinar thalamus (PT), red nucleus (RN), SN, and dentate nucleus (DN). Multi-slice 3D regions-of-interest (ROI) representing these structures were manually traced on QSM slices using SPIN (Signal Processing in NMR, SpinTech, Inc., Bingham Farms, MI, United States) by the first author (KG) with more than 5 years of relevant experience. Original magnitude and phase images were used as references to ensure accurate boundary drawings. An illustration of the 3D ROIs is given in Figure 1. Mean susceptibility values from the entire structures of both cohorts were then extracted and plotted as a function of age, also known as the global analysis. Similar to Liu et al.’s (2016) work, age-dependent susceptibility values were chosen as thresholds from the upper 95% prediction intervals based on their global analysis of 174 controls from which regional high iron (RII) content voxels were then estimated for a given structure at a given age for all the nuclei except the DN. For the DN, a similar process was performed on the global analysis established by Ghassaban et al.’s (2018) study from 81 healthy adults. Similarly, mean susceptibilities of the RII regions were calculated and plotted as a function of age, also known as the regional analysis. The global and regional analyses of both PD and HC cohorts were then superimposed on the corresponding plots introduced by Liu et al. (2016) and Ghassaban et al. (2018). Additionally, the average values were compared for both global and RII susceptibilities between the PD and HC cohorts in both hemispheres and in all DGM nuclei.

#### R2^∗^ Processing

The R2^∗^ maps were also reconstructed using the first eight echoes through a pixel-by-pixel fit to an exponential curve. The exact same 3D ROIs traced on the QSM maps were also used on R2^∗^ maps. Similar to QSM data analysis, mean R2^∗^ values were extracted from each structure and plotted as a function of age (i.e., global analysis). These values were then superimposed and visually compared to the corresponding R2^∗^-age baselines established by Li et al. (2014) for six DGM structures including the CN, GP, PUT, RN, SN, and DN. For each structure the fitted exponential regression equation and 95% confidence intervals provided by Li et al. (2014) were used to predict the mean R2^∗^ values as a function of age for the normal population. Moreover, similar to the analysis done in QSM processing, R2^∗^ values of both PD and HC cohorts were compared to each other in both hemispheres for all DGM structures.

#### Correlation Between Susceptibility and R2^∗^

With both the QSM and R2^∗^ data available and in order to assess the relationship between these two parameters in the DGM structures, a linear regression model was fitted to the mean R2^∗^ as a function of mean susceptibility in all the nuclei. To avoid any sources of bias, only the healthy cohort was included for this correlation, the results of which were compared to those of Li et al.’s (2014) study.

### Statistical Analysis

All statistical analyses were carried out using Microsoft Excel 2013 (Microsoft Corporation, Redmond, WA, United States) with a two-tailed significance level of 0.05. Two-sample *t*-test analyses were performed to compare the average global (for QSM and R2^∗^) and regional (for QSM only) values of PD and HC cohorts in both hemispheres. Furthermore, paired sample *t*-tests were performed to compare susceptibility and R2^∗^ values between the left and right hemisphere of each DGM structure.

Additionally, a comparison between the mean susceptibility values of the SN and clinical status of the PD patients was performed. The parameters to which SN global and regional susceptibility values were compared included the unified Parkinson’s disease rating scale part III (UPDRS-III) and H&Y scores as well as the disease duration.

Finally, the same clinical status parameters along with patients’ ages were used to compare a sub-group of PD patients with abnormal RII susceptibility values [i.e., higher than the corresponding upper 95% prediction interval from the susceptibility-age baseline (Liu et al., 2016)] to another sub-group whose RII susceptibilities fall within the normal ranges of the baseline.

## Results

**Figure 2:**
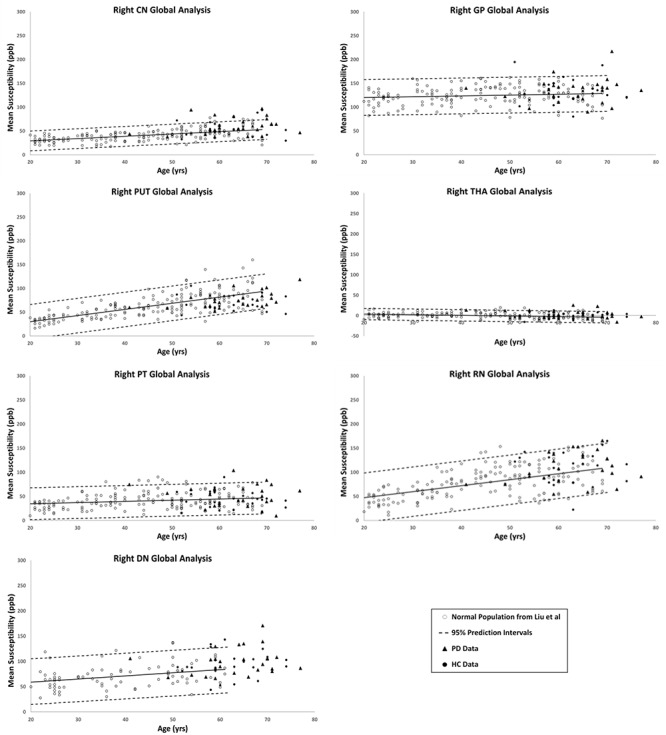
Right hemisphere global analyses of HC and PD cohorts superimposed on the susceptibility-age baselines for different deep gray matter (DGM) structures published by Ghassaban et al. (2018) for the dentate nucleus and Liu et al. (2016) for the other six nuclei. CN, caudate nucleus; GP, globus pallidus; PUT, putamen; THA, thalamus; PT, pulvinar thalamus; RN, red nucleus; DN, dentate nucleus. Hollow circles, normal baselines; solid circles, HC data from this study; triangles, PD data from this study; solid lines, linear regression models associated with the normal population; dashed lines, 95% prediction intervals associated with the normal population.

Figure 2, 3 show the QSM global and regional analyses for the right hemisphere of both groups, respectively, superimposed on the corresponding previously established normal populations. These susceptibility-age baselines were published by Ghassaban et al. (2018) (for the DN only) and Liu et al. (2016) (for the rest of the DGM structures). The SN is the only structure showing elevated susceptibility values in both global and regional analyses (see Figure 4).

**Figure 3:**
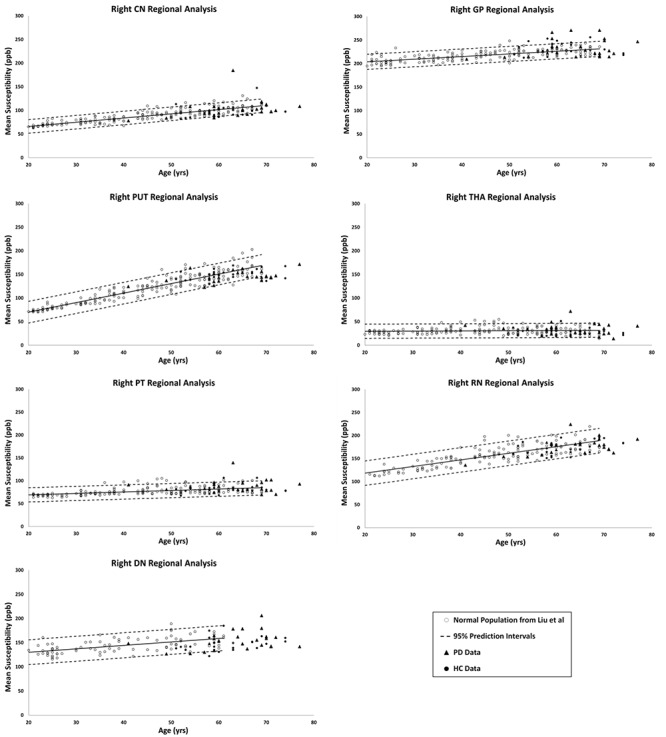
Right hemisphere regional high iron (RII) analyses of HC and PD cohorts superimposed on the susceptibility-age baselines for different DGM structures published by Ghassaban et al. (2018) for the dentate nucleus and Liu et al. (2016) for the other six nuclei. CN, caudate nucleus; GP, globus pallidus; PUT, putamen; THA, thalamus; PT, pulvinar thalamus; RN, red nucleus; DN, dentate nucleus. Hollow circles, normal baselines; solid circles, HC data from this study; triangles, PD data from this study; solid lines, linear regression models associated with the normal population; dashed lines, 95% prediction intervals associated with the normal population.

**Figure 4:**
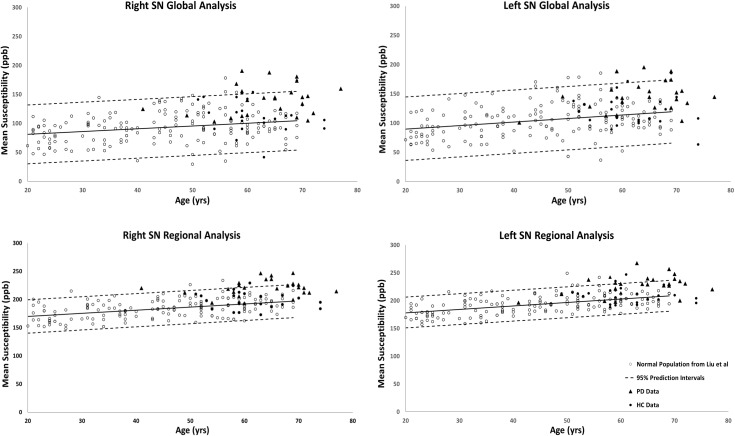
Substantia nigra global (entire structure) and regional (RII high iron) analyses of HC and PD cohorts superimposed on the susceptibility-age baselines published by Liu et al. (2016). Hollow circles, normal baselines; solid circles, HC data from this study; triangles, PD data from this study; solid lines, linear regression models associated with the normal population; dashed lines, 95% prediction intervals associated with the normal population.

The results of the two-sample and paired-sample *t*-tests comparing the susceptibility means of HC and PD cohorts within and between the two hemispheres of different DGM structures are summarized in Table 1. Only the SN showed significantly higher susceptibility values in PD patients when compared with the HC cohort, with the regional analysis (PD: 221 ± 14 ppb, HC: 200 ± 15 ppb, *p* < 0.001 for the right and PD: 235 ± 15 ppb, HC: 210 ± 15 ppb, *p* < 0.001 for the left hemisphere) revealing more prominent differences compared to those of the global analysis (PD: 140 ± 26 ppb, HC: 115 ± 29 ppb, *p* < 0.01 for the right and PD: 147 ± 27 ppb, HC: 127 ± 27 ppb, *p* < 0.01 for the left hemisphere). The SN was also the only structure showing significant differences between the two hemispheres in both cohorts using both global and regional analyses. On the other hand, in addition to the SN, significant differences were seen between the hemispheres in both PD and HC groups in the THA, RN, and DN only using the regional analysis. Among all affected nuclei, the left hemisphere showed significantly higher susceptibility values.

**Table 1:** Two-sample *t*-test statistics comparing susceptibility means (ppb) of the global and regional analyses between the two cohorts in both hemispheres.

		Global analysis			RII analysis		
Hemisphere		Right	Left	*P*-value	Right	Left	*P*-value
CN	HC	54.6 ± 6.6	52.4 ± 7.6	0.27	99.1 ± 5.6	97.3 ± 6.1	0.32
	PD	59.3 ± 6.7	54.7 ± 6.9	0.16	102.0 ± 7.8	97.7 ± 8.6	**0**.**03**
	*P*-value	0.35	0.67	NA	0.56	0.95	NA
GP	HC	133.1 ± 10.1	127.8 ± 7.8	0.15	231.8 ± 5.2	231.1 ± 6.9	0.82
	PD	133.7 ± 10.0	127.6 ± 9.9	0.11	232.3 ± 7.2	231.6 ± 7.9	0.86
	*P*-value	0.94	0.97	NA	0.92	0.92	NA
PUT	HC	68.7 ± 6.4	72.8 ± 7.0	0.25	150.5 ± 4.3	144.9 ± 5.0	0.08
	PD	75.8 ± 6.6	73.6 ± 7.8	0.32	145.1 ± 4.8	142.1 ± 6.1	0.12
	*P*-value	0.15	0.87	NA	0.19	0.10	NA
THA	HC	2.3 ± 2.8	2.8 ± 2.9	0.71	30.2 ± 3.2	36.5 ± 3.0	<**0**.**001**
	PD	3.1 ± 3.7	2.2 ± 3.7	0.59	33.3 ± 4.8	36.8 ± 3.8	<**0**.**001**
	*P*-value	0.24	0.66	NA	0.30	0.88	NA
PT	HC	45.3 ± 7.0	45.6 ± 6.9	0.90	83.3 ± 3.7	85.4 ± 2.8	0.10
	PD	51.8 ± 8.8	47.7 ± 7.2	0.12	87.5 ± 5.5	86.4 ± 4.7	0.45
	*P*-value	0.27	0.68	NA	0.22	0.74	NA
RN	HC	108.1 ± 13.0	102.9 ± 12.9	0.31	173.2 ± 5.4	177.5 ± 6.1	**0**.**03**
	PD	112.8 ± 13.1	112.2 ± 13.8	0.85	175.8 ± 6.8	179.3 ± 6.8	**0**.**01**
	*P*-value	0.61	0.36	NA	0.55	0.69	NA
SN	HC	115.4 ± 11.6	127.5 ± 10.8	**0.04**	200.1 ± 6.1	210.3 ± 5.7	<**0**.**001**
	PD	139.8 ± 10.4	147.5 ± 10.5	**0.01**	220.7 ± 5.6	234.7 ± 5.9	<**0**.**01**
	*P*-value	**< 0.01**	**< 0.01**	NA	**< 0.001**	**< 0.001**	NA
DN	HC	93.1 ± 9.7	95.3 ± 11.0	0.41	147.6 ± 5.4	155.9 ± 6.2	<**0**.**001**
	PD	99.4 ± 10.2	102.1 ± 10.8	0.41	153.7 ± 7.2	159.1 ± 7.4	<**0**.**01**
	*P*-value	0.38	0.39	NA	0.20	0.52	NA

*Numbers are reported in Mean ± 95% confidence intervals. CN, caudate nucleus; GP, globus pallidus; PUT, putamen; THA, thalamus; PT, pulvinar thalamus; RN, red nucleus; SN, substantia nigra; DN, dentate nucleus. *P*-values in bold show significant differences between the means*.

The relationship between mean global and regional susceptibility changes in the SN of PD patients and clinical status parameters (i.e., disease duration, H&Y and UPDRS-III scores) resulted in no significant correlations (all *p*-values > 0.05). However, as shown in Table 2, dividing the PD cohort in two sub-groups with normal and abnormal RII susceptibility values showed that there were significantly higher UPDRS-III scores in patients with elevated RII iron content (*p* < 0.05 in both hemispheres).

**Table 2:** Comparison of clinical status between two sub-groups of the PD cohort with normal and abnormal RII iron content in the SN.

Hemisphere	Right			Left		
	Abnormal	Normal		Abnormal	Normal
Group	iron (*N* = 10)	iron (*N* = 14)	*p*-value	iron (*N* = 10)	iron (*N* = 14)	*P*-value
Age (years)	62.5 ± 8.6	64.9 ± 7.8	0.51	63.9 ± 5.6	63.1 ± 9.7	0.80
Disease duration (years)	4.7 ± 2.8	3.8 ± 3.3	0.49	5.1 ± 3.4	3.4 ± 2.9	0.29
UPDRS-III	30.4 ± 14.3	17.8 ± 9.6	**0.03**	28.6 ± 12.9	16.6 ± 9.7	**0.03**
H&Y	1.8 ± 0.6	1.4 ± 0.5	0.17	2.0 ± 0.75	1.5 ± 0.55	0.10

*Normal and abnormal sub-groups are separated based on RII susceptibility values lower and higher than the baseline upper 95% prediction intervals, respectively. *P*-values in bold show significant differences between the means*.

**Figure 5:**
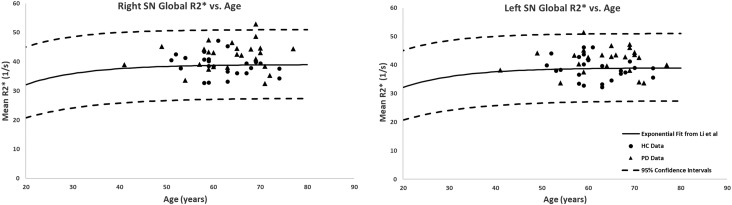
Global analysis of the R2^∗^ measurements in the SN of PD patients and healthy adults superimposed on the corresponding R2^∗^-age exponential fits provided by Li et al. (2014). Although most of the subjects fall within the 95% confidence intervals, the PD patients showed higher R2^∗^ values compared to those of the HC. Solid circles, HC data from this study; triangles, PD data from this study; solid lines, exponential regression models from the normal population (Li et al., 2014); dashed lines, 95% confidence intervals associated with the fitted curves.

The global measurements of R2^∗^ values superimposed on the corresponding age-dependent baselines published by Li et al. (2014) resulted in no abnormal values with most of the means falling within the 95% confidence intervals. The only exception was the SN of the PD patients with R2^∗^ values slightly skewed upward but still most subjects were below the upper 95% confidence interval, as shown in Figure 5. Also, the two-sample *t*-tests between PD and HC cohorts revealed significant differences in the SN in both hemispheres (PD: 42 ± 4 s^-1^, HC: 39 ± 4 s^-1^, *p* = 0.01 for the right and PD: 43 ± 4 s^-1^, HC: 39 ± 4 s^-1^, *p* = 0.01 for the left hemisphere). The paired sample *t*-tests showed no significant differences between the hemispheres for any of the structures in either cohort.

**Figure 6:**
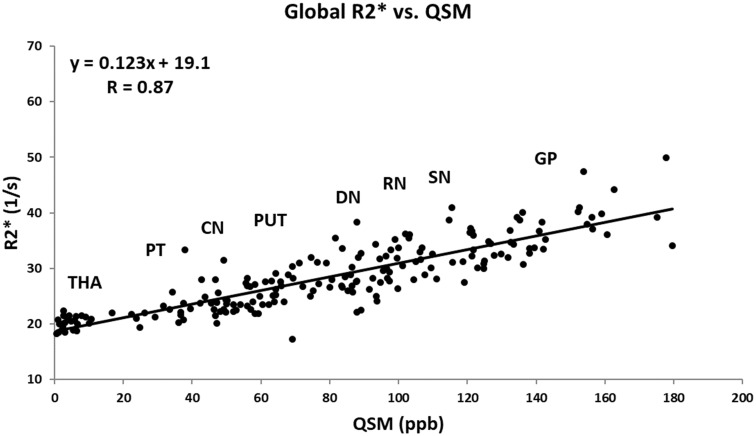
Mean R2^∗^ as a function of mean QSM in deep gray matter nuclei of the HC group fitted by a linear regression model. The data points are the average means between the two hemispheres. Based on the estimated mean values, the labels show the approximate locations around which each structure falls. The regression parameters are shown on the plot. CN, caudate nucleus; GP, globus pallidus; PUT, putamen; THA, thalamus; PT, pulvinar thalamus; RN, red nucleus; DN, dentate nucleus. Solid circles, HC data from this study; solid line, linear regression fit.

Figure 6 shows the linear regression model fitted to the R2^∗^ values as a function of the corresponding QSM values for the HC group plotted for all the nuclei included in this study. The Pearson correlation coefficient (PCC) value of 0.87 is indicative of a strong linear relationship between these two parameters. Also, the linear slope of 0.123 s^-1^/ppb is very close to the slope of 0.126 s^-1^/ppb reported by Li et al. (2014).

## Discussion and Conclusion

To date, there is no clear answer to the contributing factors in the pathogenesis of PD (Langkammer et al., 2016; Pietracupa et al., 2017). It is generally believed that the loss of neuromelanin content may initiate the process of increasing non-heme iron deposits in the midbrain which then leads to different forms of parkinsonism (Castellanos et al., 2015; Huddleston et al., 2017; Langley et al., 2017). On the other hand, there is also the hypothesis arguing that the depigmentation of the nigrosome-1 content in the posterolateral part of the SN might lead to the subsequent increase in iron deposition in this midbrain structure (Schwarz et al., 2018).

In this work, we have shown that there is an increase in iron in the SN over and above the normal increase due to age in PD patients. Although the average susceptibility value of the SN is seen to increase in the PD cohort, especially in the thresholded regions characterized by high iron content, we also note that there may, in fact, be two populations of PD patients, those that do not change iron content and those that do. For the abnormally high iron content group, there was a significantly higher UPDRS-III than the group showing normal iron content. Nevertheless, to draw a stronger conclusion will require investigating a considerably larger sample size.

The global and regional analyses in the SN of PD patients has previously been evaluated in Ghassaban et al. (2018) and Sethi et al. (2018) in which similar QSM reconstruction techniques were adopted using different scanners and field strengths. The regional analysis being more sensitive to local high iron content changes seen in the SN in this study is in accordance with their findings. This validates the consistency and reliability of using gradient echo imaging and the use of the threshold-based QSM reconstruction for data from different MR manufacturers’ systems.

The comparison between the two hemispheres revealed significantly higher iron deposition in the left hemispheres of both PD and HC cohorts in the THA, RN, SN, and DN in the regional (RII) analysis and only in the SN for the global analysis. However, the largest effects in terms of absolute shifts in susceptibility were seen in the SN where the differences were on the order of 20 ppb compared to all other cases where the differences are on the order of less than 8 ppb. This is consistent with Liu et al.’s (2016) study where they showed that, except in the SN, small but significant differences between the hemispheres of all DGM structures would vanish if other sources of error (in their case excluding one slice from the top and/or bottom of the structures or changing the definition of RII from upper 95 to 99% prediction interval of the global analysis) were taken into account when measuring the susceptibility values. However, simulation results for the SN show that QSM has a systematic error of roughly 12 ppb in the SN due to streaking artifacts generated by the QSM reconstruction (Haacke et al., 2015). Additionally, low spatial frequency undulations seen in QSM techniques could also lead to asymmetry in the brain (Haacke et al., 2015). Therefore, by taking into consideration all these sources of systematic error, the asymmetries seen in the SN may disappear as well. Another QSM study in which lateral asymmetry was found in the SN of PD patients was done by Azuma et al. (2016) where the mean susceptibility was seen to be significantly higher in the more affected hemisphere compared to that of the less affected hemisphere although they had much larger errors compared to our data due in part to the distribution of iron and the small sample size. They also do not account for the other sources of systematic error mentioned above which may again remove any remaining small differences between left and right values of iron in the SN.

Evaluating R2^∗^ maps led to two major findings: first, in accordance with the literature, iron content characterized by R2^∗^ values was seen to be higher in the SN of PD patients compared to the healthy group. Second, even though these differences were statistically significant, considerably lower *p*-values from QSM results (both between the two groups and between the hemispheres within each group) showed higher sensitivity and reliability of susceptibility-based techniques to pick up more subtle changes in brain iron. Additionally, the error analysis from both of these iron quantification techniques reveals that the variability of measurements associated with QSM is considerably less compared to that of R2^∗^ (Feng et al., 2018), especially in the high iron content (RII) region. Despite the fact that R2^∗^ correlates well with QSM measures of iron content, R2^∗^ measurements (especially from a limited number of echo times) is more prone to noise. One major advantage of QSM is that it is in theory independent of echo time, but the SNR of the R2^∗^ maps depends critically on the echo time (Haacke et al., 2015). Practically an echo time of roughly 20 ms is enough to give excellent susceptibility maps and in this case with 8 echoes excellent measurements of the DGM is possible.

Another key finding in this work that validates previous results is the tightness (higher *r*^2^-values) of the iron growth with age in the different DGM structures in the regional iron content measures. The fact that regional changes are much tighter than global changes opens the door to a better separation of patient types, specifically in terms of separating high iron content patients from normal iron content patients. Averaging over all patients, especially in the global analysis, will reduce the shift in the mean iron content and may be the reason that some of the previous studies failed to show increased iron content in the SN (Yan et al., 2018). RII iron content may provide a new means to evaluate the role of regional changes in iron deposition. For example, it is believed that the loss of neuromelanin in the nigrosome-1 territory of the SN pars compacta leaves behind MR-visible iron (Zecca et al., 2004). Localizing where this high iron content occurs anatomically may help to answer this question.

Further, in this work, the ability to separate the high and normal RII iron content patients using QSM data led to the finding that there are in fact group differences in UPDRS-III scores. Similarly, by using R2^∗^, Pesch et al. (2019) found a correlation between iron content and UPDRS-III scores in the SN of 35 PD patients. However, their R2^∗^ measurements were not corrected for age. Since R2^∗^ has been seen to increase as a function of normal aging, (Li et al., 2014) it is imperative to take this factor into account in iron quantification studies.

There are a number of limitations to this study. First, the number of samples is small and a much larger population should be studied to best demonstrate the presence of two groups of PD patients. Second, no clinical phenotypes were taken into consideration while analyzing imaging data. Third, ROI tracings were done manually which might have induced some unwanted errors when demarcating different DGM nuclei especially around the edges of the structures; this source of error, however, gets substantially reduced when thresholded low susceptibility values are excluded in the regional analysis. Nonetheless, the undesired errors associated with manual ROI tracing in the global analysis could be effectively minimized by using atlas-based automated DGM segmentation techniques (Li et al., 2019). Finally, the fairly low in-plane resolution used in the GRE sequence made it difficult to evaluate the sub-structures, especially the SN pars compacta whose abnormally high iron deposition is believed to be correlated with neuromelanin degeneration in the midbrain (Castellanos et al., 2015; Huddleston et al., 2017; Langley et al., 2017). Higher spatial resolution is recommended in future studies for this purpose.

In conclusion, the increase in iron in the SN in some PD patients is higher than the normal range in HC as found in both regional and global analyses. Using RII content may provide a means to separate two populations of PD patients; one with and one without iron increases in the SN. Separating the PD population into two groups may prove useful in understanding the etiology of the disease as well as monitoring the disease progression.

## Ethics Statement

This study was carried out in accordance with the recommendations of the “ethics committee of Ruijin Hospital, Shanghai Jiao Tong University School of Medicine, Shanghai, China” with written informed consent from all subjects. All subjects gave written informed consent in accordance with the Declaration of Helsinki. The protocol was approved by the “ethics committee of Ruijin Hospital, Shanghai Jiao Tong University School of Medicine, Shanghai, China.”

## Author Contributions

NH, FY, EMH, PH, and SC conceived the research project. NH, EMH, and FY organized the research project. KG and NH executed the research project. KG and SKS designed the statistical analysis. KG and SKS executed the statistical analysis. NH and EMH critically reviewed the statistical analysis. KG wrote the first draft of the manuscript. NH and EMH critically reviewed the manuscript.

## Conflict of Interest Statement

SKS and EMH have part time positions in Magnetic Resonance Innovations, Inc. The remaining authors declare that the research was conducted in the absence of any commercial or financial relationships that could be construed as a potential conflict of interest.
